# Supplementary material for: Pervasive Sharing of Causal Genetic Risk Factors Contributes to Clinical and Molecular Overlap between Sjögren’s Disease and Systemic Lupus Erythematosus
Source: Int J Mol Sci. 2023 Sep 22;24(19):14449. doi: 10.3390/ijms241914449 (PMC10572278; doi:10.3390/ijms241914449)
Supplement: Supplementary file 1 [file ijms-24-14449-s001.zip › Supplementary Figures.pdf]

Supplemental Material for *Pervasive sharing of causal genetic risk factors contributes to clinical and molecular overlap between Sjögren's Disease and Systemic Lupus Erythematosus*

Analysis 1: The casual effect of SjD variants on SLE in European ancestry GWAS

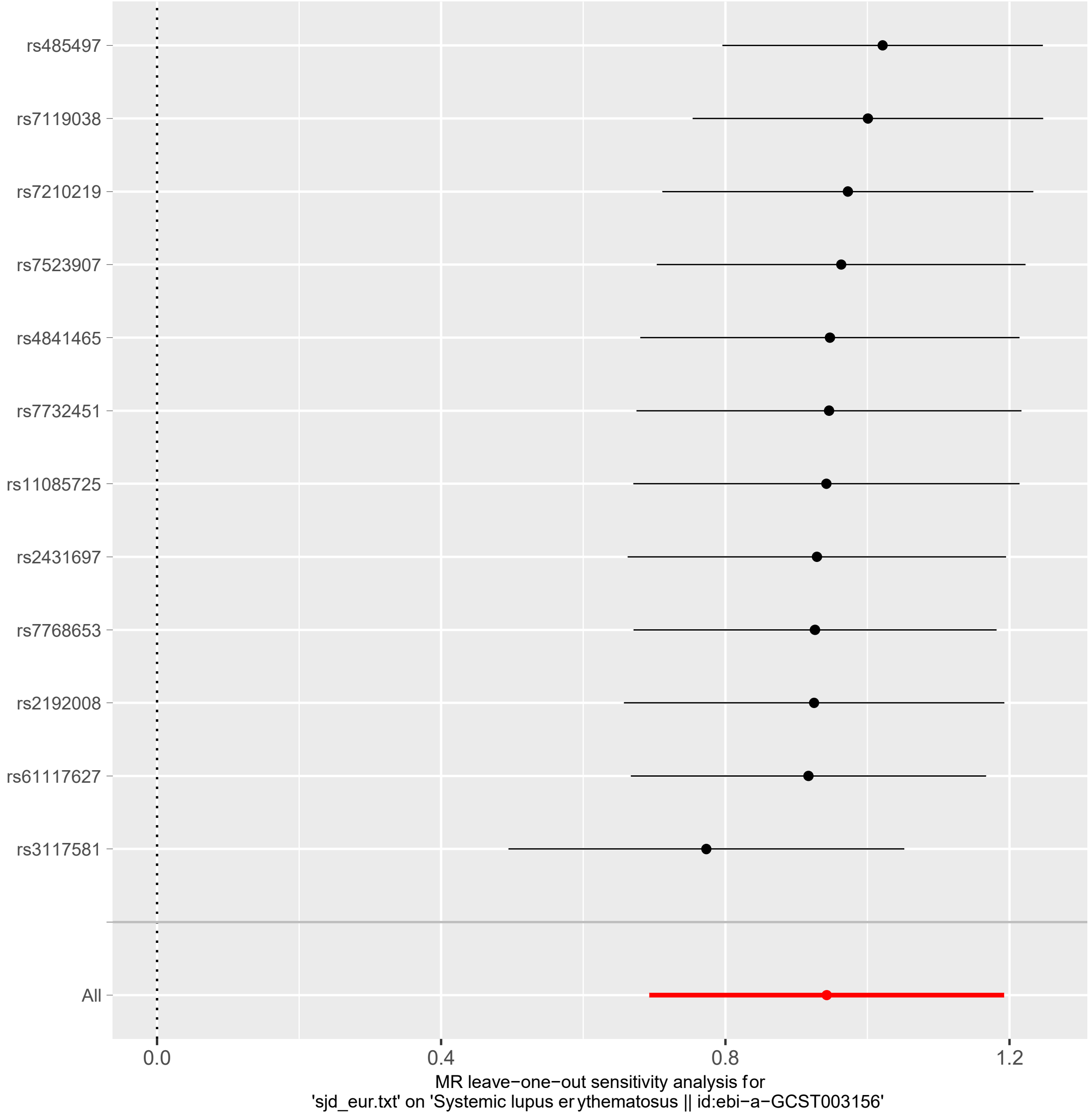

Figure S1. Leave-one out sensitivity Analysis reveals a stable causal relationship between Sjögren's Disease and Systemic Lupus Erythematosus in a GWAS of persons with European ancestry Forest plot of the aggregate (inverse-variance weighted) MR effect sizes (beta+/-se) of SjD risk variants on SLE in the Bentham J., et al. GWAS with the indicated variant left out.

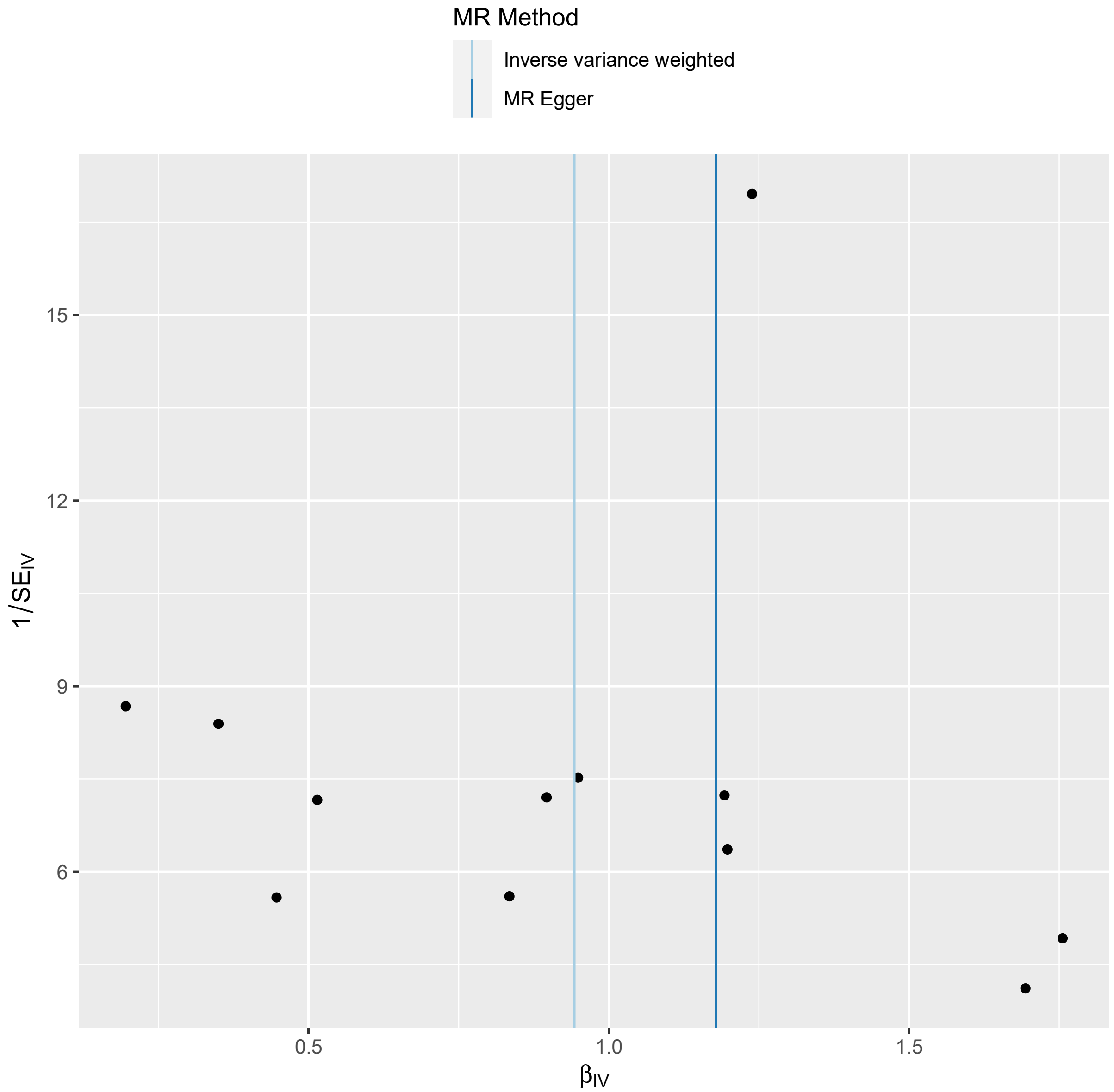

Figure S2. Funnel Plot of effects of SjD variants in SLE GWAS performed in a cohort of persons with European ancestry. Shown is the inverse of the standard error plotted as a function of the effect sizes for SjD risk variants on SLE in the Bentham J., et al. GWAS. Values lower on the y-axis are less precise. The wider the distribution, the greater the heterogeneity, which can arise from horizontal pleiotropy. Asymmetry indicates directionality, which can bias MR methods. MR-Egger adjusts for such directional horizontal pleiotropy.

Analysis 2: The casual effect of SLE variants on SjD in European ancestry GWAS.

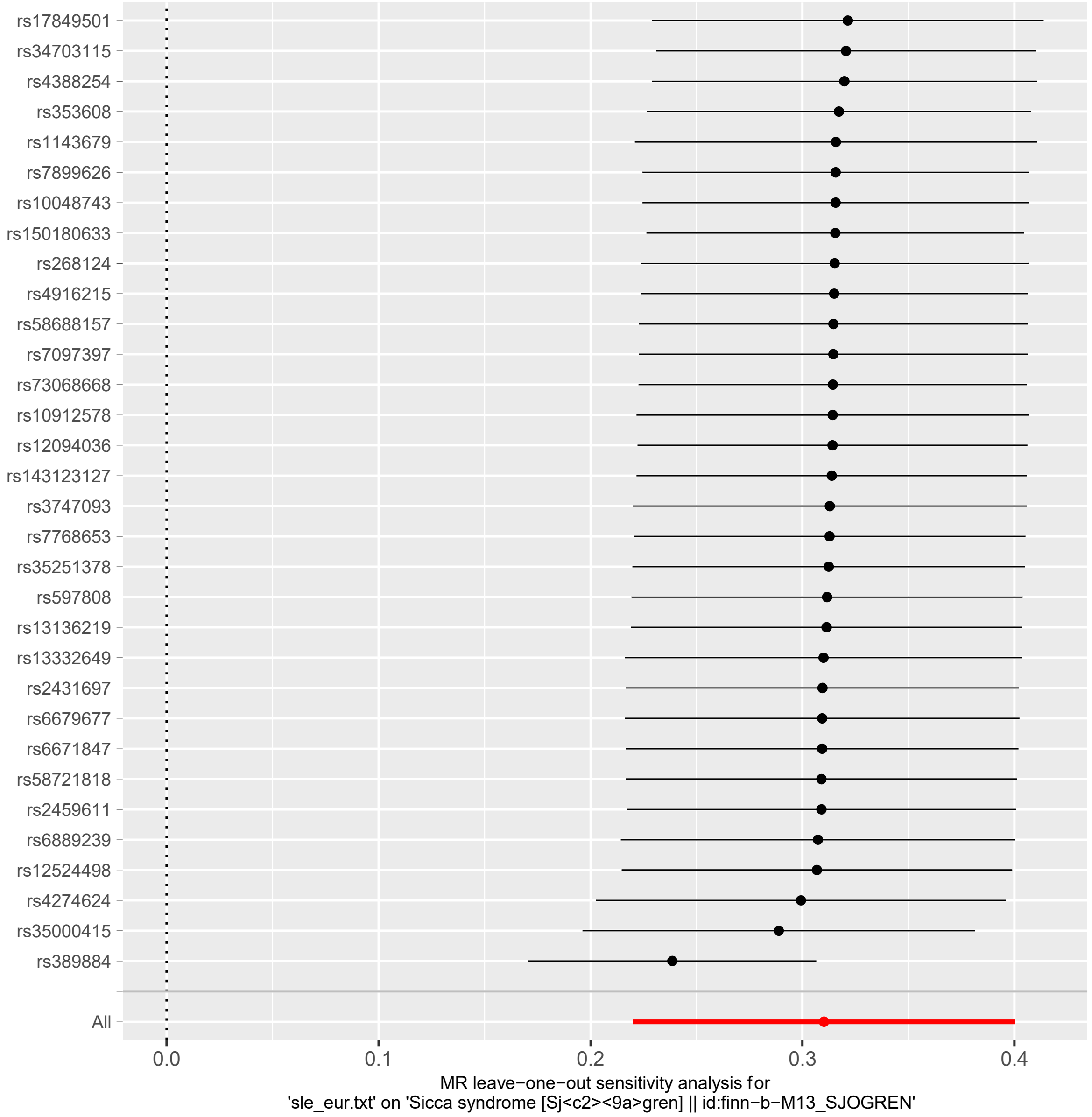

Figure S3. Leave-one out sensitivity Analysis reveals stable causal relationship between SLE and SjD in a GWAS of persons with European ancestry. Forest plot of the aggregate (inverse-variance weighted) MR effect sizes (beta+/-se) of SLE risk variants on SjD in the Finngen M13_Sjogren GWAS with the indicated variant left out.

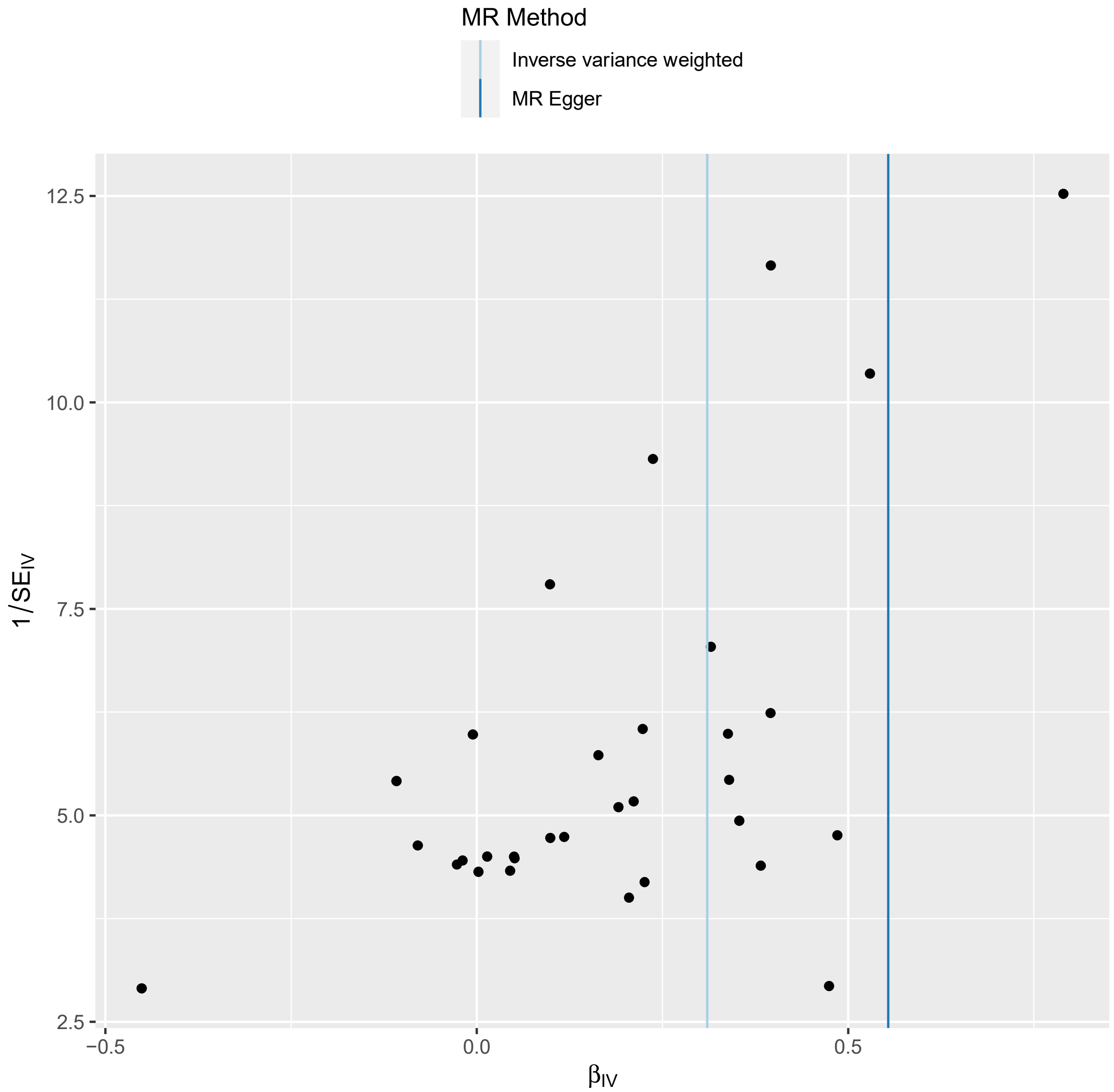

Figure S4. Funnel Plot of effects of SLE variants on SjD GWAS performed in a cohort of persons with European ancestry. Shown is the inverse of the standard error plotted as a function of the effect sizes for SLE risk variants on SjD in the Finngen M13_Sjogren GWAS. Values lower on the y-axis are less precise. The wider the distribution, the greater the heterogeneity, which can arise from horizontal pleiotropy. Asymmetry indicates directionality, which can bias MR methods. MR-Egger adjusts for such directional horizontal pleiotropy.

Analysis 3: The casual effect of SjD variants on SLE in East Asian ancestry GWAS.

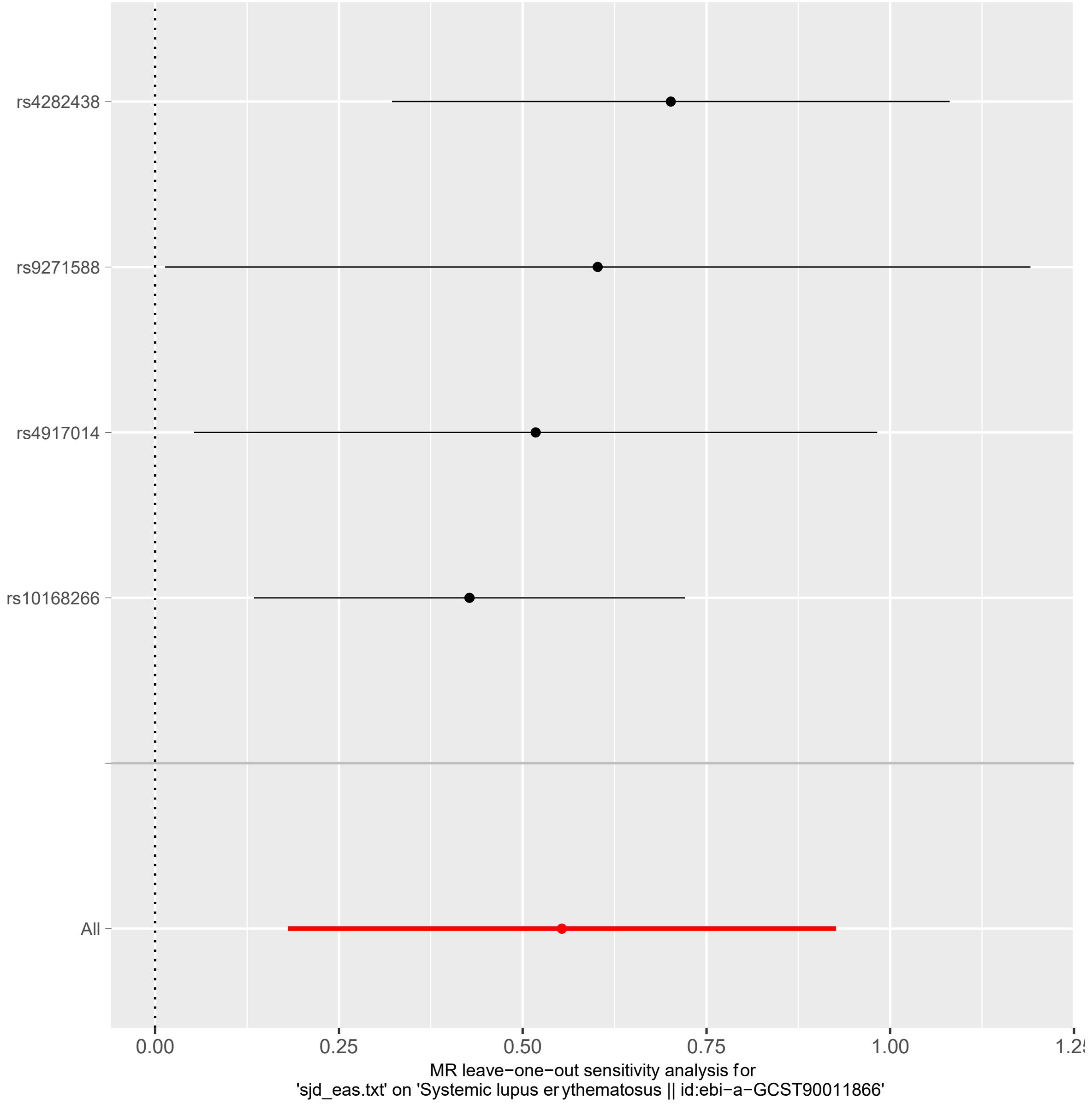

Figure S5. Leave-one out sensitivity Analysis reveals stable causal relationship between SjD and SLE in a GWAS of persons with East Asian ancestry. Forest plot of the aggregate (inverse-variance weighted) MR effect sizes (beta+/-se) of SjD risk variants on SLE in the Wang YF, et al. GWAS with the indicated variant left out.

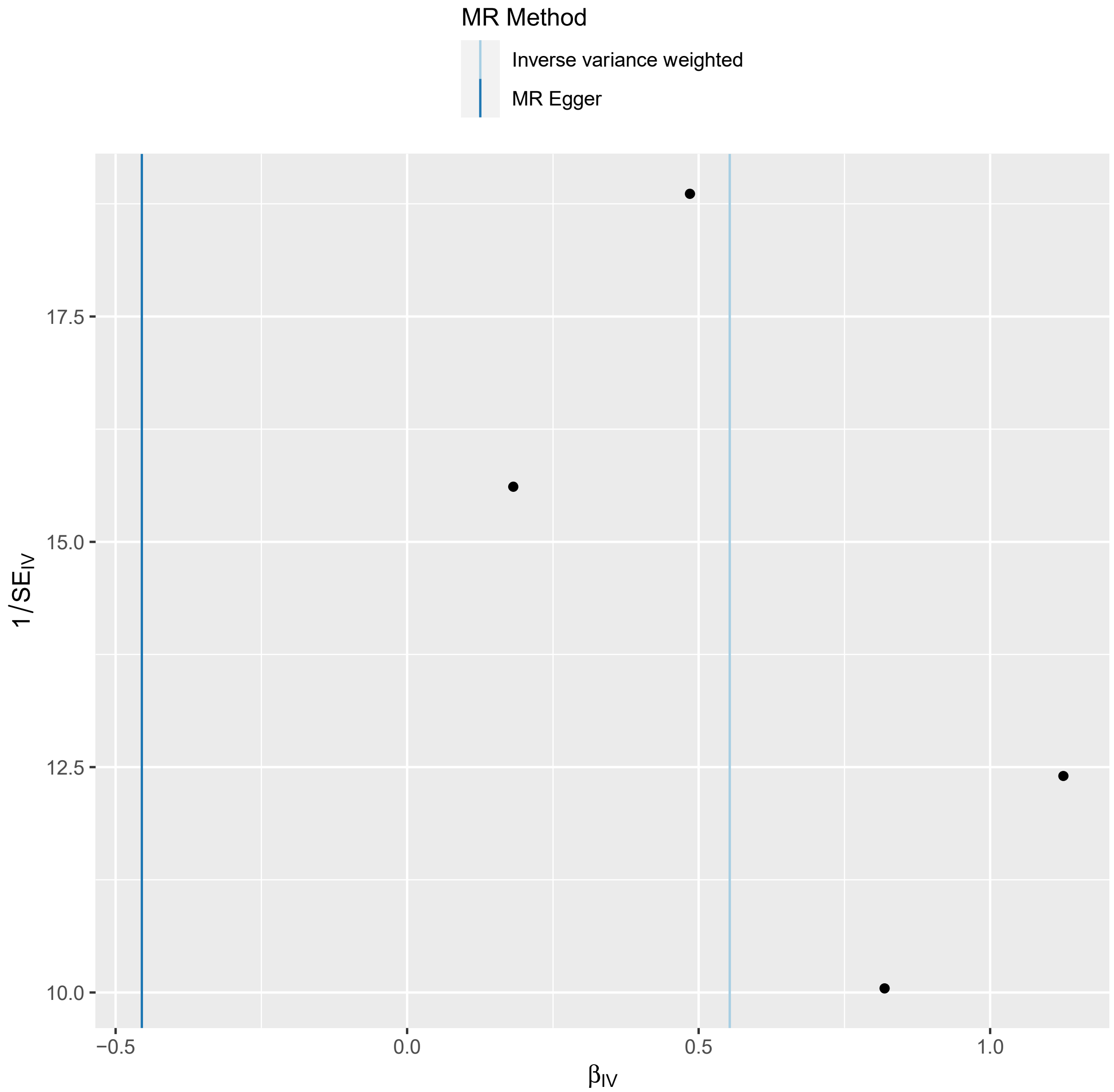

Figure S6. Funnel Plot of effects of SjD variants identified in SLE GWAS performed in a cohort of persons with East Asian ancestry. Shown is the inverse of the standard error plotted as a function of the effect sizes for SjD risk variants on SLE in the Wang, YF, et al. SLE GWAS. Values lower on the y-axis are less precise. The wider the distribution, the greater the heterogeneity, which can arise from horizontal pleiotropy. Asymmetry indicates directionality, which can bias MR methods. MR-Egger adjusts for such directional horizontal pleiotropy.

Analysis 4: The casual effect of SLE variants from East Asian ancestry GWAS on SjD in European ancestry GWAS.

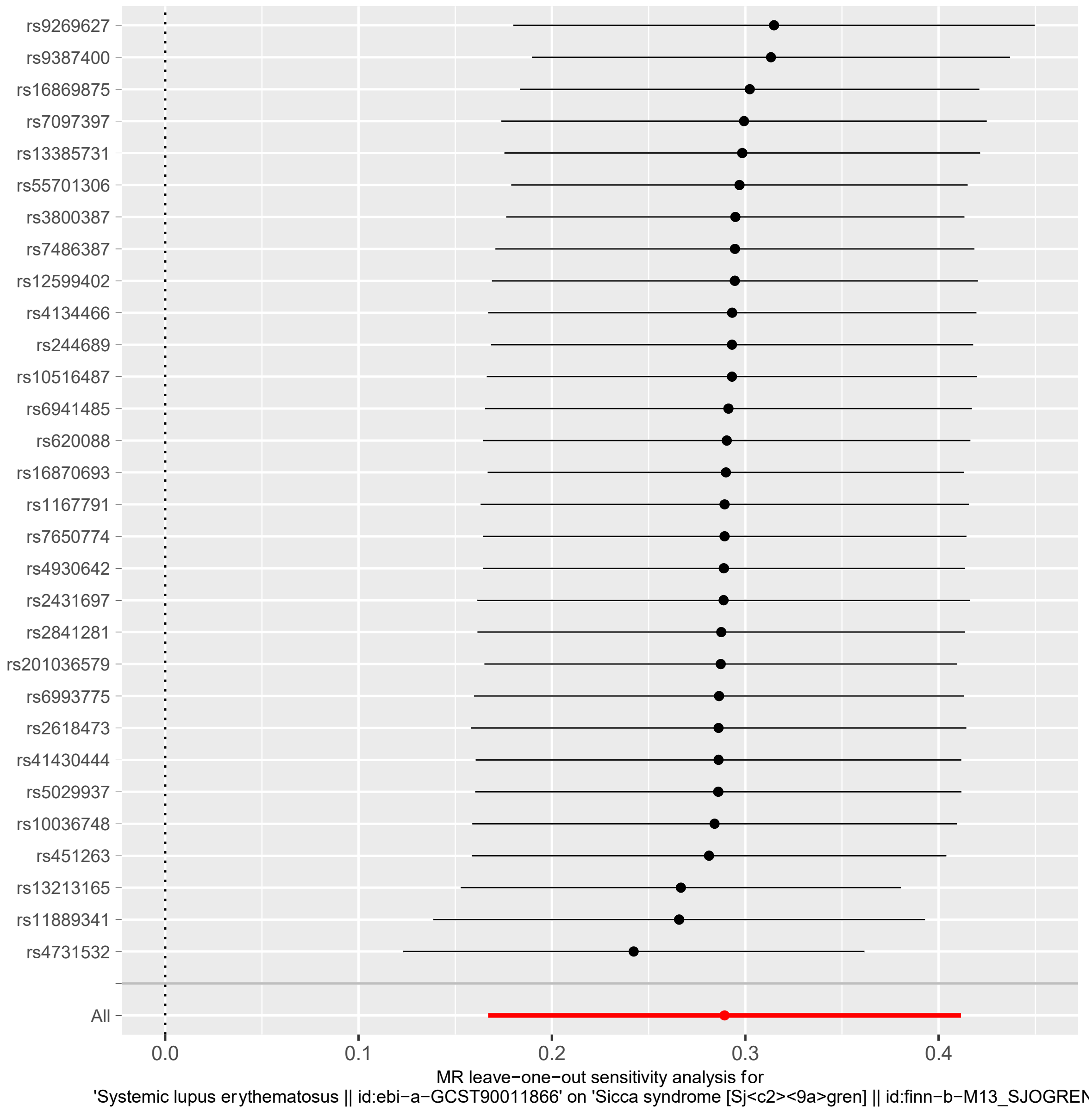

Figure S7. Leave-one out sensitivity Analysis reveals stable causal relationship between SLE variants identified in GWAS of persons with East Asian ancestry on SjD in a GWAS of persons with European ancestry. Forest plot of the aggregate (inverse-variance weighted) MR effect sizes (beta+/-se) of SLE risk variants on SjD in the Finngen M13_Sjogren GWAS with the indicated variant left out.

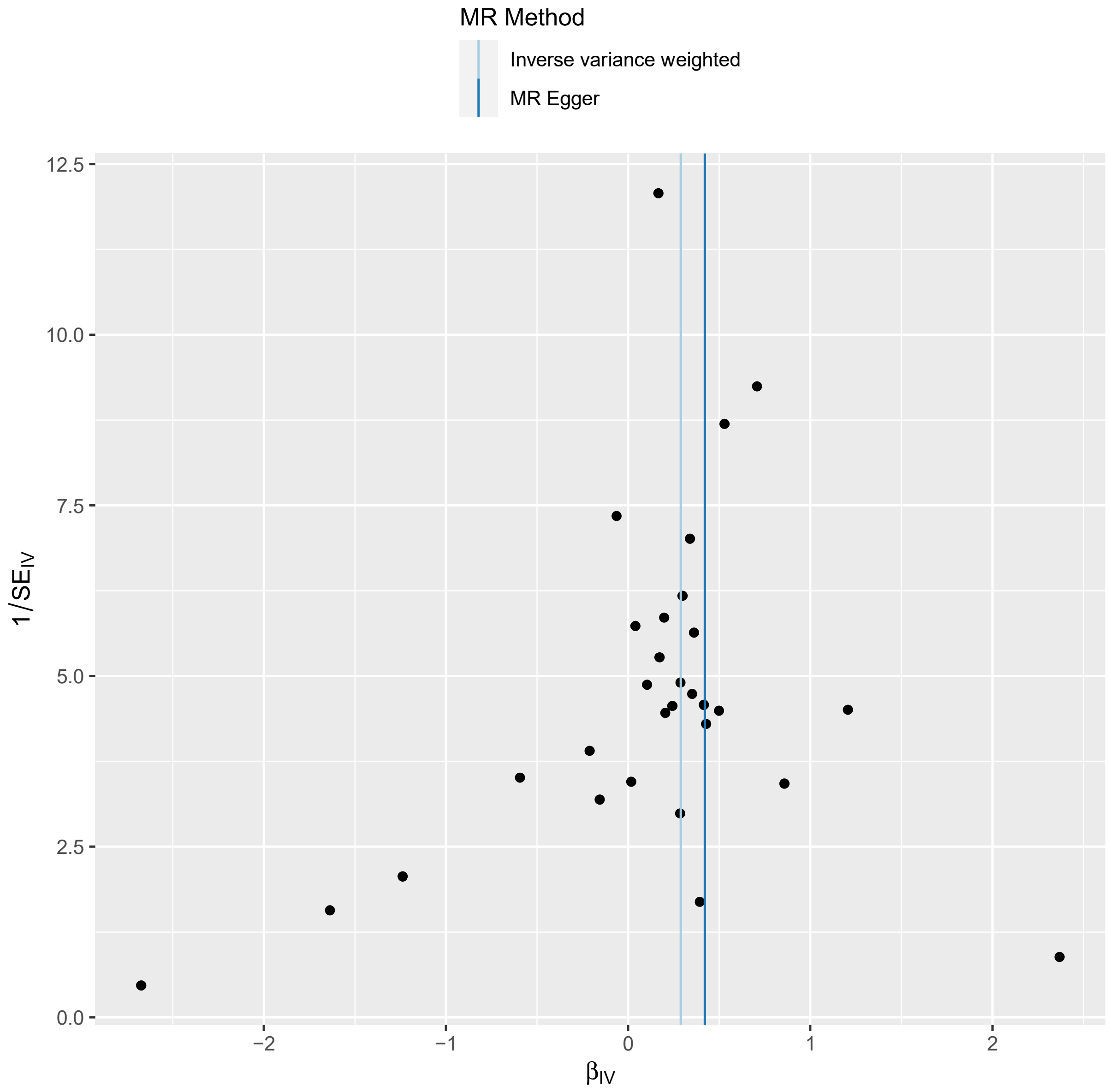

Figure S8. Funnel Plot of effects of SjD variants identified in SLE GWAS performed in a cohort of persons with East Asian ancestry. Shown is the inverse of the standard error plotted as a function of the effect sizes for SLE risk variants on SjD in the Finngen M13_Sjogren GWAS. Values lower on the y-axis are less precise. The wider the distribution, the greater the heterogeneity, which can arise from horizontal pleiotropy. Asymmetry indicates directionality, which can bias MR methods. MR-Egger adjusts for such directional horizontal pleiotropy.
