# Supplementary material for: Pervasive Sharing of Causal Genetic Risk Factors Contributes to Clinical and Molecular Overlap between Sjögren’s Disease and Systemic Lupus Erythematosus
Source: Int J Mol Sci. 2023 Sep 22;24(19):14449. doi: 10.3390/ijms241914449 (PMC10572278; doi:10.3390/ijms241914449)
Supplement: Supplementary file 1 [file ijms-24-14449-s001.zip › Supplementary Note.pdf]

Supplemental Note:

Method for synthesizing and comparing published GWAS results for Sjogren Syndrome and SLE

Part 1: make a list of the published genome wide-significant regions from the EBI GWAS catalog for Sjogren syndrome

1. Go to EBI/NHGRI GWAS catalog: [https://www.ebi.ac.uk/gwas/]
2. Search for “Sjogren syndrome”
3. Select:

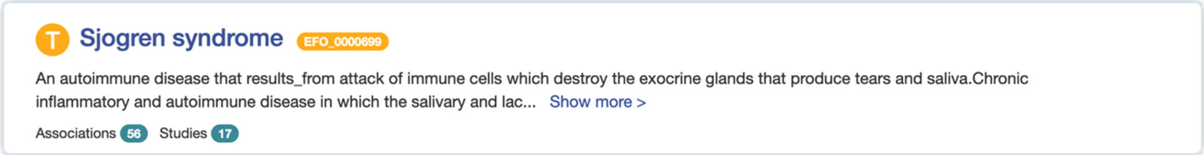

4. Scroll down and select the “export data” button

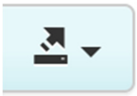

5. Click on CSV and open resulting file in Excel

6. Sort location by chromosome and position

7. Remove markers that lack mapping information in the EBI GWAS catalog.

8. Remove variants with P-value > 5E-8

9. Define regions. Starting with the first variant row, go through all the rows and increase the region number each time a marker that is a genome wide-significant (P< 5E-8) marker > 250,000 bases away is observed or a marker on a different chromosome is observed.

Part 2: collate the putative causal genes for each region from the published open targets genetics L2G pipeline results

1. Select the open targets genetics website for trait of interest [https://genetics.opentargerts.org]
2. For each GWAS study of the trait of interest in open targets genetics and eah region in the table from part 1, add the gene listed in L2G column (“Genes prioritized by our locus-to-gene-model with score ≥ 0.5”) to the “Putative Causal Gene” column

Part 3: Enter the putative causal genes into string-db.org and to putative polygenic causal gene network.

1. Go to string-db.org

2. Click search
3. Click multiple proteins on the left hand side
4. Remove commas and ensure that each putative causal gene is on a single line
5. Enter this list of genes in the "list of names" box

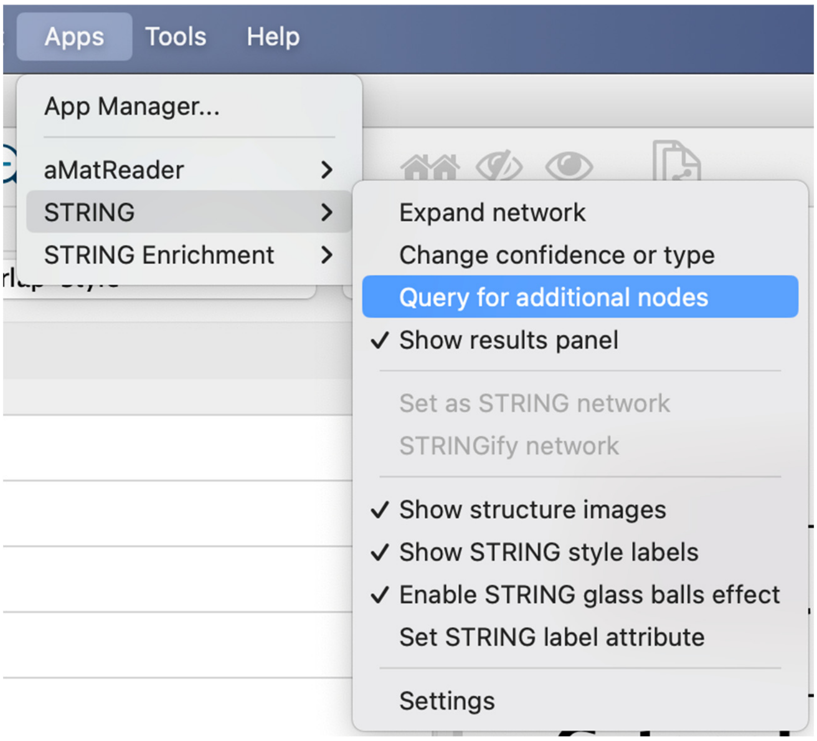

6. Select "Homo sapiens" as organism
7. Search
8. Review gene names and descriptions to ensure that he correct mapping occurred.
9. Click continue
10. To merge networks.
    a. Open network of interest in Cytoscape
    b. Click exports and send network to Cytoscape
    c. Click merge networks in Cytoscape

Part 4: Merge Sjogren syndrome's networks with SLE network

Monogenic and polygenic SLE networks were adopted from "Polygenic autoimmune disease risk alleles impacting B cell tolerance act in concert across shared molecular networks in mouse and in humans (https://www.frontiersin.org/articles/10.3389/fimmu.2022.953439/full).

1. Open "Monogenic and polygenic human SLE risk gene networks" (Figure 1) in Cytoscape from Ndex Bio (https://www.ndexbio.org/viewer/networks/aa310899-c7de-11ec-b397-0ac135e8bacf)
2. Select the names of the causal genes from the putative causal Sjogren genes excel file and copy
3. In Cytoscape, on the opened "Monogenic and polygenic human SLE risk gene networks," select "Apps." If the STRING app is not installed, install it in the App Manager. Once installed, select "Query for additional nodes"

4. In the pop-up window, paste the previously copied list of putative causal Sjogren genes, and then select import. Do not re-layout network.
5. Genes that did not already exist in the monogenic and polygenic SLE risk network will populate. Annotate network to differentiate, between genes that overlap with the SLE and Sjogren network and the genes that overlap with the monogenic and polygenic SLE network, prioritizing those that overlapped with SLE and Sjogren.

References:
[EBI GWAS Catalog]
Sollis, E.; Mosaku, A.; Abid, A.; Buniello, A.; Cerezo, M.; Gil, L.; Groza, T.; Güneş, O.; Hall, P.; Hayhurst, J.; et al. The NHGRI-EBI GWAS Catalog: Knowledgebase and Deposition Resource. Nucleic Acids Res. 2023, 51, D977–D985. https://doi.org/10.1093/nar/gkac1010.

https://www.ebi.ac.uk/gwas/

[EBI GWAS Catalog Manual]
https://www.ebi.ac.uk/gwas/docs

[L2G model]
Mountjoy, E., Schmidt, E.M., Carmona, M. et al. An open approach to systematically prioritize causal variants and genes at all published human GWAS trait-associated loci. Nat Genet 53, 1527–1533 (2021). https://doi.org/10.1038/s41588-021-00945-5

[L2G Overview & Documentation]
https://genetics-docs.opentargets.org/our-approach/prioritising-causal-genes-at-gwas-loci-l2g

[Open Targets Genetics Portal & Documentation]
https://genetics.opentargets.org/ https://genetics-docs.opentargets.org/

[STRING Database]
Szklarczyk D*, Gable AL*, Nastou KC, Lyon D, Kirsch R, Pyysalo S, Doncheva NT, Legeay M, Fang T, Bork P‡, Jensen LJ‡, von Mering C‡.
The STRING database in 2021: customizable protein–protein networks, and functional characterization of user-uploaded gene/measurement sets .
Nucleic Acids Res. 2021 Jan 8;49(D1):D605-12.PubMed

Szklarczyk D, Gable AL, Lyon D, Junge A, Wyder S, Huerta-Cepas J, Simonovic M, Doncheva NT, Morris JH, Bork P‡, Jensen LJ‡, von Mering C‡.
STRING v11: protein-protein association networks with increased coverage, supporting functional discovery in genome-wide experimental datasets.

Nucleic Acids Res. 2019 Jan; 47:D607-613.PubMed

[STRING Database website]

https://string-db.org/

[STRING & Cytoscape tutorial youtube channel from Lars Juhl Jensen]
https://www.youtube.com/c/LarsJuhlJensen

[Cytoscape]
Shannon P, Markiel A, Ozier O, Baliga NS, Wang JT, Ramage D, Amin N, Schwikowski B, Ideker T. Cytoscape: a software environment for integrated models of biomolecular interaction networks Genome Research 2003 Nov; 13(11):2498-504

[Cytoscape website, documentation and manual]
https://cytoscape.org/what_is_cytoscape.html https://github.com/cytoscape/cytoscape-tutorials/wiki https://manual.cytoscape.org/en/stable/

[Web-based hypergeometric distribution calculator]
https://systems.crump.ucla.edu/hypergeometric/index.php

from the Graeber lab:
https://systems.crump.ucla.edu/
